# Prevalence of complementary and alternative medicine (CAM) use in Brazil

**DOI:** 10.1186/s12906-020-2842-8

**Published:** 2020-02-13

**Authors:** Patricia de Moraes Mello Boccolini, Cristiano Siqueira Boccolini

**Affiliations:** 1 Faculdade de Medicina de Petrópolis, FASE, Rio de Janeiro, Brazil; 2 Instituto de Informação e Comunicação Científica e Tecnológica em Saúde, Fiocruz, Rio de Janeiro, Brazil

**Keywords:** Complementary therapies, Traditional medicine, Epidemiological surveys, Unified health system

## Abstract

**Background:**

The use of medicinal plants or other alternative practices can be the only therapeutic resources for many communities and ethnic groups, especially in developing countries. In Brazil, the Ministry of Health incorporated Complementary and Alternative Medicine (CAM) as a public health policy since 2006. The aim of this study was to evaluate the prevalence of CAM use in Brazil.

**Methods:**

This was a cross-sectional study performed as an epidemiological survey, with data from the National Health Survey, 2013 that evaluated a sample of adult Brazilians (18+ years old). The outcome was the use of CAM therapies, such as acupuncture, homeopathy, medicinal plants and herbal medicines in the last 12 months. We employed a logistic regression model (CI 95%) to evaluate the chances of CAM use.

**Results:**

The prevalence of CAM use in Brazil was 4.5%. The subjects with higher chances to use CAM were: women (AOR = 1.42), aged > 40 years (AOR = 1.64), with higher educational levels (AOR = 2.35), and residents at North (AOR = 2.02) and South (AOR = 1.67) regions of Brazil, all with *p*-value < 0.001. According to the socioeconomic status, subjects from upper classes had higher chances to use acupuncture and homeopathy when compared to the other classes, and individuals from lower classes had higher chances to use medicinal plants and herbal medicines. Almost half of all individuals reporting CAM use did so outside the health care system. The Brazilian Unified Health System (SUS) was the least used funding for CAM when compared to other types of funding.

**Conclusions:**

We recommend that the Ministry of Health invests in capacity building for health professionals who work with CAM, providing structure for those practices in health services, increasing the access of CAM therapies for SUS users, and improving the registering of information about those therapies, encouraging the use of CAM by the Brazilian population.

## Background

The use of medicinal plants for health treatment, cure or even disease prevention can be considered one of the oldest forms of medical practice [1]. Even today, the use of medicinal plants or other alternative practices can be the only therapeutic resources for many communities and ethnic groups, especially in developing countries [2–4].

Since the Alma Ata Declaration in the late 1970s, the World Health Organization (WHO) has stated its position, encouraging the safe use of medicinal plants for health treatments, considering that approximately 80% of the world’s population use those resources as primary health care [5]. An important fact is that developing countries residents are the ones that most often use these practices, and those countries have 67% of the world’s plant species [5].

The Traditional Medicine Program was created by the WHO in the late 1970s and, over the following years, they reinforced their commitment to stimulate the development of public policies to include such practices into the health systems of all member states [6]. The Traditional Medicine Program is also named Complementary or Alternative Medicine in countries where the health system is still based on allopathic medicine or where Traditional Medicine has not been incorporated into the national health systems [7].

Studies indicate that the use of herbal products, minerals or even non-medication practices, such as acupuncture, are beneficial to human health, provided that the individual has knowledge about their purpose, risks, benefits and even possible harmful interactions [8–11].

The use of complementary medicine has been increasing in developed countries for disease prevention and health promotion purposes [12–14]. Among developing countries, the use of complementary medicine is possibly attributed to its accessibility [15, 16], which may be strongly associated with the traditional belief and knowledge systems, since complementary medicine may be the only accessible source of health care in those settings [7].

Brazil has stood out in this scenario as one of the countries that have specific policies for complementary medicine use. A National Ordinance (MS-971/2006) was published in 2006, which included in the Brazilian Unified Health System (SUS) the Traditional Chinese Medicine/Acupuncture, Homeopathy, Medicinal Plants and Herbal Medicines [17], named Complementary and Integrative Practices in Brazil (or PIC, in Portuguese). In 2017 and 2018, two new National Ordinances (MS 849/2017 and MS 702/2018) were released, which included new complementary medicine therapies into the National Policy for Complementary and Integrative Practices (or PNPIC, in Portuguese), expanding the use of these services and aiming the safe, respectful, equitable and effective access to those practices by the population that relies only on SUS [18]. Complementary medicine is an important practice and the prevalence of its use remains unknown within the health care system of the country, even with efforts to consolidate and broaden the scope of National Policy for Complementary and Integrative Practices [19].

## Methods

The aim of this study was to evaluate the prevalence of Complementary and Alternative Medicine (CAM) use in Brazil with data from the National Health Survey (PNS). PNS was a population-based cross-sectional study designed to evaluate the health conditions and lifestyle of the Brazilian adult population, conducted between 2013 and 2014. The complex sample process included stratification by Brazilian region, capitol and metropolitan area, and other areas, with conglomerates in three stages: census sector units as primary sample cluster; households as secondary sample unit; and adults (18 years old and more) living in the household as a tertiary sample unit. The sample size considered the desired level of accuracy for the estimation of some indicators at different disaggregation levels and population groups [20]. The study was performed by the Oswaldo Cruz Foundation and the Brazilian Ministry of Health, in partnership with the Brazilian Institute of Geography and Statistics (*Instituto Brasileiro de Geografia e Estatística*, IBGE).

The questionnaire was subdivided into three parts. The first two were answered by a resident of the household and included questions about the household characteristics and the socioeconomic and health status of all residents. The individual questionnaire was answered by a resident of 18 years or older, selected with equal probability from all adult residents of the household and focused on morbidity and lifestyles. The complete questionnaire of the “Pesquisa Nacional de Saúde” can be found in Portuguese at https://www.pns.icict.fiocruz.br/index.php?pag=proposicao, with CAM-related questions at section “J” [21].

The survey sample was designed to represent the Brazilian adult population (18+ years old), and had a complex design that comprised 81,254 households, of which 69,994 were occupied. In the end, 64,348 home interviews were conducted, having 60,202 interviews with the selected resident, as detailed in Souza-Junior et al. [20] (2015) and Damacena et al. [22] (2015).

The outcome evaluated in this study was the use of CAM in the last 12 months, defined according to Ordinance MS 971/2006 as the use of acupuncture, homeopathy, medicinal plants and herbal medicines, without the distinction among those two last options. The PNS/2013 questionnaire did not include a question specifically directed to the use of traditional Chinese medicine.

The outcome was obtained by a sequence of 5 questions, starting with: “In the last 12 months, has/have _____ used any complementary and alternative practice, i.e. treatment such as acupuncture, homeopathy, medicinal plants and herbal medicines, etc.?”, considering a binary answer (yes / no). Subsequently, to qualify this outcome, the treatments used (acupuncture, homeopathy, medicinal plants and herbal medicines, and others) were discriminated. The funding source for the CAM was built by deriving 3 sequential and non-excluding questions: “Was this treatment covered by any health insurance?”; “Did you pay anything for this treatment?”; and finally, “Was this treatment provided by the Brazilian Unified Health System (SUS)?”

The obtained final variable (funding model) was categorized as: SUS, private health insurance, private (when using neither health insurance nor SUS, but payment for the service was reported), and “not funded” (when none of the three previous situations applied).

To assess the individual sociodemographic characteristics, the following variables were considered: region of residence (Southeast, South, Midwest, Northeast, North); gender (male; female); age group (18–39; 40–59; and 60 years and over); educational level (incomplete elementary school; complete high school and higher); skin color/ethnicity (white; non-white); and socioeconomic status, according to the Brazilian Criterion – ABEP (2019) [23], divided into classes A + B (higher), C (intermediate), D + E (lower).

Finally, we employed a logistic regression model, having the use of any CAM as an outcome, and sociodemographic characteristics (sex, age, educational level, Brazilian regions, race, and socioeconomic index) as confounders. Another three logistic regression models were estimated, having specific CAM use (homeopathy, acupuncture and herbal and medicinal plants) as outcomes and employing the same confounders. Adjusted Odds Ratios (AOR) were obtained for each variable.

To avoid the design effect, the complex sample design was considered in all analyses stages, having a confidence interval of 95% and *p*-value< 0.05 for all variables.

## Results

In Table 1 we can observe that the prevalence of any Complementary and Alternative Medicine (CAM) use in Brazil was 4.5%. The North and South regions had the highest prevalence of CAM use. It was observed that women, the population over 40 years old and those with higher educational level were the ones who most often use any CAM. According to the socioeconomic status variable, it was observed that social class C was the one that least used any CAM.

**Table 1:** Prevalence of Complementary and Alternative Medicine (CAM) use, according to sociodemographic characteristics, PNS 2013^e^

Sociodemographic variables	Prevalence %^a^	95% Confidence Interval ^b^
Brazilian regions		
Southeast	3.8	(3.2–4.5)
South	6.1	(5.1–7.3)
Midwest	4.1	(3.5–4.9)
Northeast	4.1	(3.5–4.8)
North	7.0	(5.8–8.5)
Gender		
Male	3.7	(3.3–4.2)
Female	5.2	(4.7–4.7)
Age (years)		
18 to 39 years	3.4	(3.0–3.9)
40 to 59 years	5.5	(4.9–6.2)
60 years or older	5.4	(4.7–6.1)
Educational level^c^		
Up to Incomplete Elementary School	4.2	(3.6–4.9)
Up to Complete High School	3.3	(3.0–3.7)
From Incomplete College/University	8.1	(7.2–9.1)
Ethnicity/skin color		
White	4.7	(4.3–5.2)
Non-white	4.3	(3.7–4.9)
Socioeconomic status^d^		
Higher (A + B)	5.6	(5.0–6.2)
Intermediate (C)	3.1	(2.8–3.5)
Lower (D + E)	5.4	(4.5–6.5)
Total prevalence	4.5	(4.1–4.9)

^a^ Prevalence considering the complex sample design

^b^ 95% confidence interval considering the complex sample design

^c^ The variable education categories “illiterate and incomplete elementary school” were aggregated

^d^ The Brazil Criterion variable

^e^ Sample of individuals aged 18 years and older who answered the 2013 National Health Survey individual questionnaire

In Table 2 we can observe the prevalence of CAM therapy use according to sociodemographic characteristics, with the Brazilian North region residents the ones that most often used medicinal plants and herbal medicine, the Southeast region residents using more acupuncture, whereas the South region residents most often used homeopathy. Medicinal plants and herbal medicines were widely used by both genders, and women use all therapies more often than men. Individuals aged 60 and over most often use acupuncture and medicinal and herbal medicine.

**Table 2:** Prevalence of Complementary and Alternative Medicine (CAM) use, according to sociodemographic characteristics, PNS 2013^e^

Sociodemographic Variables	Prevalence of CAM use^a^ (95%CI)^b^			
	Acupuncture	Homeopathy	Medicinal Plants and Herbal Medicines	Others
Brazilian regions				
Southeast	1.5 (1.3–1.9)	0.6 (0.5–0.9)	1.3 (0.9–1.9)	0.3 (0.2–0.4)
South	1.0 (0.7–1.4)	1.0 (0.7–1.6)	3.6 (2.8–4.7)	0.4 (0.2–0.8)
Midwest	0.7 (0.5–0.9)	0.7 (0.5–1.0)	2.5 (2.0–3.2)	0.2 (0.1–0.4)
Northeast	0.3 (0.2–0.4)	0.2 (0.1–0.3)	3.5 (2.9–4.2)	0.1 (0.1–0.2)
North	0.1 (0.1–0.3)	0.6 (0.4–0.8)	6.2 (4.9–7.7)	0.1 (0.1–0.3)
Gender				
Male	0.6 (0.5–0.9)	0.5 (0.3–0.6)	2.4 (2.1–2.8)	0.2 (0.1–0.3)
Female	1.3 (1.1–1.5)	0.7 (0.6–0.8)	2.9 (2.6–3.4)	0.3 (0.2–0.4)
Age (years)				
18 to 39 years	0.5 (0.4–0.7)	0.5 (0.4–0.7)	2.1 (1.8–2.5)	0.2 (0.1–0.3)
40 to 59 years	1.3 (1.0–1.7)	0.7 (0.5–0.9)	3.1 (2.6–3.6)	0.4 (0.2–0.5)
60 years or older	1.4 (1.1–1.7)	0.5 (0.3–0.6)	3.4 (2.9–4.0)	0.2 (0.1–0.3)
Educational level^c^				
Up to Incomplete Elementary School	0.3 (0.2–0.5)	0.3 (0.2–0.4)	3.5 (2.9–4.1)	0.1 (0.1–0.2)
Up to Complete High School	0.8 (0.6–0.1)	0.4 (0.3–0.6)	1.9 (1.6–2.2)	0.2 (0.2–0.4)
From Incomplete College/University	3.2 (2.6–3.9)	1.8 (1.4–2.3)	2.4 (1.9–2.9)	0.7 (0.4–1.1)
Ethnicity				
White	1.5 (1.2–1.7)	0.8 (0.7–1.0)	2.1 (1.8–2.4)	0.4 (0.3–0.5)
Non-white	0.5 (0.4–0.7)	0.4 (0.3–0.5)	3.2 (2.8–3.8)	0.2 (0.1–0.2)
Socioeconomic status^d^				
Higher (A + B)	2.2 (1.8–2.6)	1.3 (1.0–1.6)	1.6 (1.3–1.9)	0.5 (0.3–0.7)
Intermediate (C)	0.5 (0.4–0.7)	0.3 (0.2–0.4)	2.1 (1.9–2.4)	0.2 (0.2–0.3)
Lower (D + E)	0.1 (0.0–0.3)	0.2 (0.1–0.2)	5.1 (4.1–6.2)	0.1 (0.0–0.1)
Total prevalence	1.0 (0.8–1.1)	0.6 (0.5–0.7)	2.7 (2.4–3.0)	0.3 (0.2–0.3)

^a^ Prevalence considering the complex sample design

^b^ 95% confidence interval considering the complex sample design

^c^ The variable education categories “illiterate and incomplete elementary school” were aggregated

^d^ The Brazil Criterion variable

^e^ Sample of individuals aged 18 years and older who answered the 2013 National Health Survey individual questionnaire

In Table 3 we analyzed the prevalence of the type of funding for CAM according to the socio-demographic characteristics. SUS was the most often used funding system for CAM in the North and Northeast regions, when compared to the other regions. Women were the ones who most often used CAM through SUS. Regarding the age group, individuals aged 40 to 59 years old were the ones who most often used CAM funded by SUS. Individuals with up to incomplete elementary school were the ones who most often used CAM funded by SUS, when compared to other educational levels.

**Table 3:** Prevalence of Complementary and Alternative Medicine (CAM) by funding type, according to the sociodemographic characteristics, PNS 2013^e^

Sociodemographic Variables	Prevalence of CAM use ^a^ (95%CI) ^b^ by Funding Type			
	SUS	Private Health Insurance	Private	Not funded
Brazilian regions				
Southeast	5.8 (3.7–8.8)	20.2 (15.9–25.2)	44.7 (39.3–50.2)	29.4 (24.8–34.5)
South	5.3 (3.1–9.0)	13.0 (9.5–17.6)	36.2 (30.7–42.1)	45.5 (39.2–51.9)
Midwest	5.5 (3.5–8.6)	13.5 (9.4–19.0)	36.4 (30.3–42.9)	44.6 (37.7–51.8)
Northeast	6.5 (4.3–9.8)	5.2 (3.8–7.2)	21.8 (17.7–26.4)	66.5 (62.0–70.7)
North	7.6 (4.5–12.7)	2.1 (1.1–4.2)	23.3 (18.0–29.5)	67.0 (60.1–73.2)
Sex				
Male	5.4 (3.6–7.9)	10.9 (8.0–14.6)	27.5 (23.3–32.0)	53.3 (51.0–61.4)
Female	6.5 (5.0–8.4)	13.6 (11.4–16.2)	38.7 (35.4–42.2)	41.2 (38.2–44.3)
Age (years)				
18 to 39 years	4.4 (3.2–6.2)	12.4 (9.4–16.0)	32.1 (27.8–36.6)	51.1 (46.6–55.7)
40 to 59 years	7.2 (5.1–10.3)	12.5 (10.0–15.6)	34.8 (30.2–39.7)	45.5 (40.9–50.1)
60 years or older	6.6 (4.1–10.3)	12.9 (9.3–17.5)	37.3 (31.8–43.2)	43.3 (37.4–49.3)
Educational level^c^				
Up to Incomplete Elementary School	7.8 (5.7–10.6)	4.1 (2.9–5.8)	20.6 (16.6–25.2)	67.5 (62.8–71.9)
Up to Complete High School	7.1 (5.0–10.0)	12.2 (9.0–16.4)	37.8 (33.1–42.7)	42.9 (38.5–47.5)
From Incomplete College/University	2.3 (1.2–4.5)	25.9 (21.0–31.6)	52.1 (45.9–58.4)	19.6 (15.2–24.9)
Ethnicity				
White	4.6 (3.0–6.9)	18.4 (15.2–22.0)	43.2 (39.2–47.3)	33.8 (30.0–37.9)
Non-white	7.5 (5.8–9.8)	6.7 (5.2–8.6)	25.4 (21.8–29.4)	60.4 (56.2–64.4)
Socioeconomic status^d^				
A + B	2.7 (1.7–4.3)	25.2 (21.0–29.9)	50.3 (45.2–55.3)	21.8 (18.3–25.8)
C	9.5 (6.8–13.2)	7.5 (5.3–10.6)	33.1 (28.6–37.9)	49.9 (45.2–54.6)
D + E	7.1 (4.8–10.3)	0.6 (0.2–1.4)	14.1 (10.8–18.2)	78.3 (73.6–82.3)
Total	6.1 (4.8–7.6)	12.5 (10.7–14.7)	34.3 (31.5–37.2)	47.1 (44.2–50.0)

^a^ Prevalence considering the complex sample design

^b^ 95% confidence interval considering the complex sample design

^c^ The variable education categories “illiterate and incomplete elementary school” were aggregated

^d^ The Brazil Criterion variable

^e^ Sample of individuals aged 18 years and older who answered the 2013 National Health Survey individual questionnaire

According to the socioeconomic status, class C (intermediate) was the one that most often used CAM funded by SUS, when compared to other classes. CAM funded by private health insurance was most commonly used by those who live in the Southeast and South regions, when compared to other regions. Women use private health insurance for CAM funding more often than men. The use of private health insurance for CAM funding is similar across the three age groups assessed in the study (Table 3).

Regarding the educational level, individuals with incomplete College/University education use CAM more frequently through private health insurance, when compared to other educational levels. According to the socioeconomic status, classes A + B were the one that most often uses CAM through private health insurance when compared to the other classes. The private funding for CAM use was more prevalent in the Southeast, South and Southeast regions, when compared to the other regions (Table 3).

Table 4 shows the prevalence of the funding model used according to the CAM therapies. In SUS, the most commonly used CAM therapies were medicinal plants and acupuncture. For the private health insurance model, acupuncture and homeopathy are the most common ones. For the private model, the use of medicinal plants and herbal medicines and acupuncture were the most frequent ones. As for the not-funded model of access to CAM, the most often used were medicinal plants and herbal medicines.

**Table 4:** Prevalence of Complementary and Alternative Medicine (CAM) use, according to funding, PNS 2013 ^c^

CAM Funding	Prevalence of CAM use ^a^ (95%CI) ^b^			
	Acupuncture	Homeopathy	Medicinal Plants and Herbal Medicines	Others
SUS	28.0 (17.8–41.1)	15.1 (9.1–24.0)	48.7 (37.7–59.9)	8.1 (4.7–13.7)
Health Insurance	59.0 (50.7–66.9)	24.0 (17.4–32.2)	6.8 (4.6–10.1)	10.1 (6.1–16.3)
Private	29.1 (24.9–33.8)	21.9 (17.9–26.5)	39.7 (34.6–45.0)	9.3 (6.5–13.1)
Not funded	4.9 (3.4–7.0)	2.9 (1.7–5.2)	90.5 (87.6–92.7)	1.7 (1.0–2.9)

^a^ Prevalence considering the complex sample design

^b^ 95% confidence interval considering the complex sample design

^c^ Sample of individuals aged 18 years and older who answered the 2013 National Health Survey individual questionnaire

Considering the complete model (use of one or more type of CAM), women’s, and older persons with higher educational levels had higher chances of using all CAM’s when compared with men, lower and middle educational level and persons with less than 39 years old. The exception was regarding homeopathy, with no differences in age categories. No differences in race were observed, but the richest persons had higher chances to use homeopathy and acupuncture than the poorest, and the poorest had higher chances of using herbal medicines and medicinal plants as CAM. Regarding the Brazilian regions, important differences were observed in total and type of CAM used: the South and North Regions residents had higher chances of using one or more types of CAM; the Southeast residents higher chances to use Acupuncture as CAM, and lowest chances to use medicinal plants than the rest of the Brazilian regions, having the North and South Regions the highest chances of using medicinal plants. Homeopathy had similar chances to be used as CAM in all Brazilian regions, with the exception of North, with less than half the chances (Table 5).

**Table 5:** Factors associated with the use of Complementary and Alternative Medicine, PNS 2013^e^

Prevalence of CAM use ^a^ (95%CI) ^b^ by sociodemographic variables				
	Acupuncture	Homeopathy	Medicinal Plants and Herbal Medicines	CAM total
	aOR (95%CI)	aOR (95%CI)	aOR (95%CI)	aOR (95%CI)
Brazilian regions				
Southeast	1.00	1.00	1.00	1.00
South	0.67 (0.44–0.99)^p^	1.51 (0.90–2.52)	3.01 (1.99–4.54) ^p^	1.67 (1.31–2.13) ^p^
Midwest	0.53 (0.36–0.76) ^p^	1.24 (0.80–1.93)	1.75 (1.12–2.73) ^p^	1.09 (0.85–1.40)
Northeast	0.31 (0.20–0.49) ^p^	0.45 (0.27–0.74) ^p^	1.97 (1.22–3.18) ^p^	1.08 (0.82–1.41)
North	0.18 (0.09–0.37) ^p^	1.59 (0.97–2.60)	3.73 (2.27–6.13) ^p^	2.02 (1.49–2.74) ^p^
Gender				
Male	1.00	1.00	1.00	1.00
Female	1.95 (1.35–2.81) ^p^	1.47 (1.02–2.12) ^p^	1.23 (1.04–1.46) ^p^	1.42 (1.24–1.61) ^p^
Age (years)				
18 to 39 years	1.00	1.00	1.00	1.00
40 to 59 years	2.37 (1.63–3.47) ^p^	1.26 (0.81–1.96)	1.51 (1.26–1.81) ^p^	1.64 (1.40–1.92) ^p^
60 years or older	2.97 (2.03–4.34) ^p^	1.03 (0.67–1.58)	1.49 (1.22–1.81) ^p^	1.62 (1.37–1.91) ^p^
Educational level ^c^				
Up to Incomplete Elementary School	1.00	1.00	1.00	1.00
Up to Complete High School	1.60 (0.85–3.01)	0.87 (0.47–1.64)	0.89 (0.72–1.09)	0.99 (0.82–1.20)
From Incomplete College/University	4.22 (2.05–8.69) ^p^	2.41 (1.22–4.77) ^p^	1.50 (1.04–2.18) ^p^	2.35 (1.79–3.07) ^p^
Ethnicity/skin color				
White	1.29 (0.87–1.91)	1.22 (0.78–1.89)	0.80 (0.65–0.99) ^p^	0.99 (0.85–1.16)
Non-white	1.00	1.00	1.00	1.00
Socioeconomic status ^d^				
Higher (A + B)	1.98 (1.22–3.20) ^p^	2.99 (1.71–5.22) ^p^	0.70 (0.51–0.95) ^p^	1.27 (1.01–1.59) ^p^
Intermediate (C)	1.00	1.00	1.00	1.00
Lower (D + E)	0.36 (0.12–1.05)	0.70 (0.40–1.22)	2.04 (1.57–2.65) ^p^	1.77 (1.41–2.23) ^p^

^a^ Prevalence considering the complex sample design

^b^ 95% confidence interval considering the complex sample design

^c^ The variable education categories “illiterate and incomplete elementary

school” were aggregated”

^d^ The Brazil Criterion variable

^e^ Sample of individuals aged 18 years and older who answered the 2013 National Health Survey individual questionnaire

^p^ *p*-value < 0.05 compared with reference category

## Discussion

More than seven million adult Brazilian individuals reported the use of Complementary and Alternative Medicine (CAM) in 2013, which represents a prevalence of 4.5%. A great heterogeneity was observed regarding the types of CAM practices used between the Brazilian regions, socioeconomic strata and personal characteristics, but, in general, women and older individuals residents in the South and North regions of Brazil had higher chances of using CAM, with the richest population with more chances to use homeopathy and acupuncture, and the poorest using medicinal plants and herbal medicines. The prevalence of CAM use in the overall population can range from 10 to 75% worldwide [24]. There is no previous national estimate for the use of CAM in Brazil, but the comparison of data from different countries shows a variability in prevalence: in the United States the prevalence of CAM use was 38%, whereas in Norway it was 12.6% and in Czech Republic it was 76% [24–26].

Considering the World Regions, the Asian and Western Pacific countries have more consolidated policies and programs, with herbal medicines being regulated and registered [27]. Surveys from South Korea [28], Japan [29] and Singapore [30] observed similar CAM use prevalence among them (75, 76 and 76%, respectively), higher than Malaysia [31], Thailand [32] and Australia [33], with CAM use prevalence of 52%, 55.6, and 68.9%, respectively.

Those differences may be due both to methodological issues and to the sociodemographic diversity of those practices in the countries. In Czech Republic survey the question about CAM use considered the last 30 days prior the interview [26], while in United States [34] and Norway [35] the time period of CAM use considered the last 12 months before the interview. Another study from Australia [33] observed the prevalence of CAM use of 68.9% in the last 12 months, while if the same population were asked if CAM therapy was prescribed by a health professional the prevalence dropped to 44%.

Considering the sociodemographic characteristics, the present study showed that women, individuals with higher educational levels and those living in the North and South regions had higher chances to use any type of CAM. Similar findings were found in other studies, with different settings and cultures, suggesting the existence of intra-country sociodemographic patterns regarding the use of CAM [36–38]. In United States, for example, CAM use was more frequent among women, richest individuals and with higher educational level [38].

Our study has found important intra-country patterns, with regional differences regarding all and specific CAM therapies, even after adjusting for individual characteristics. Brazil is a continental size country and is divided into five different macro-regions - North, Northeast, Middle West, Southeast and South - with marked economic and health disparities among them [39]. The South and Southeast region are wealthier, highly urbanized, and more populated, with better access to the health care system, while North and Northeast regions have less urbanized population, with lower income and worst healthcare access: most of the Amazon forest is located in Northern states. Middle West region is less populated, embracing most of Brazilian crops and cattle in this area, with healthcare access worse than Southeast and South regions, but better than North and Northeast [39, 40].

Those regional sociodemographic differences might explain why residents from different regions have such different patterns in CAM use, with Southeast residents using acupuncture more than residents of other regions, and Southern and Northern residents with higher chances of using medicinal plants and herbal medicine than Southeast inhabitants.

Considering the CAM therapies, medicinal plants and herbal medicines were the most often used. Considering sociodemographic characteristics according CAM therapies, it was observed that individuals who more often use medicinal plants and herbal medicines had lower socioeconomic status, incomplete elementary school, were non-white, over 60 years old and lives in the North region (the poorest one in Brazil), which is in accordance with the WHO statement that poorest populations with lower access to health services use CAM more regularly, especially medicinal plants outside the scope of medical services [41].

Acupuncture is the second most often used CAM in Brazil: individuals with higher socioeconomic status, higher educational level and residents from the Southeast region use acupuncture more often. A representative survey of the overall population carried out in Australia found similar results regarding the socioeconomic characteristics of acupuncture users [42]. Since 2002, the WHO has indicated acupuncture for the treatment of some acute and chronic diseases because it is considered safe, with minimal and non-toxic side effects, in addition to its low cost, and the use of low-technology equipment [43].

The Brazilian Ministry of Health has also prioritized acupuncture as a therapeutic conduct provided by SUS, with the approval of the National Policy for Integrative and Complementary Practices. However, our study showed that acupuncture remains scarcely used in SUS, compared to its use provided by private health insurance.

Homeopathy were the least commonly CAM therapies used in Brazil when compared to the others. It was observed that the ones who most often use homeopathy are individuals with higher educational levels, with higher socioeconomic status and funded by health insurance. There are few Brazilian studies about the prevalence of CAM use: a cross-sectional study carried out in the city of Montes Claros, in the state of Minas Gerais, found a prevalence of 8.9% of CAM use in the population, with a low prevalence of acupuncture and homeopathy [44]. However, this study considered only the CAM therapies that involved consultation with a specialist [44]. According to the WHO data, in developing countries such as Brazil, homeopathy is not among the most commonly used CAM [45].

The use of homeopathy is more prevalent in Europe, especially Germany, the United Kingdom and Switzerland, where this therapy is even covered by the mandatory basic health insurance [46, 47]. Almost 50% of all individuals who reported using CAM did so outside the health system, referred to in the study as “not-funded”, i.e., all CAM that were not performed through SUS, private health insurance or even through payments made to private professionals. It was observed that the “not-funded” type was the most frequent in all Brazilian regions (especially in the North and Northeast regions), in both genders, for all age ranges, among those with a low educational level and, especially, among the lower social classes. Medicinal plants and herbal medicines are the most commonly used CAM types through the “not-funded” therapies.

According to some studies, the knowledge about plants usually passes down through generations within a family, with emphasis on the role of the mother and friends, indicating the strong sociocultural component of this practice and the scarce use of this CAM within the health care system [48, 49]. The choice of using medicinal plants is often not only due to the availability of the local flora, but also because it is considered healthier and more natural, and because it does not have the adverse effects of many allopathic medicines [48, 50]. Although SUS provides health care to two-thirds of the Brazilian population [40], it was the least used type of funding for CAM, when compared to the others. Within the scope of SUS, medicinal plants and herbal medicines, followed by acupuncture, were the most commonly CAM used. Older individuals, those with a lower educational level, lower socioeconomic status and non-whites are the ones who most often use CAM within the SUS.

In order to guarantee comprehensive care within the SUS scope, the incorporation of CAM into the public health care network, mainly in Primary Care, was promulgated as a decree by the Ministry of Health in 2006 to support and promote all traditional knowledge associated with the use of medicinal plants and other CAM, thus establishing the National Policy for Complementary and Integrative Practices in Health, which legitimizes, regulates and incorporates these practices. In 2017 and 2018, the Ministry of Health published Ordinances MS 849/2017 and MS 702/2018, respectively, increasing to twenty-nine the number of CAM offered and recognized by the Brazilian Unified Health System. By promoting a diverse range of therapeutic resources, the National Policy for Complementary and Integrative Practices provides increased care and health service coverage, encourages innovative alternatives that can contribute to the sustainable development of communities by encouraging social participation [51].

Considering the methodological aspects for CAM assessment, the prevalence variation observed on international and national studies may be associated with the questionnaires and approaches used. Some studies have recommended that the researches must specify the estimated period of CAM use (the most commonly used is a retrospective twelve-month period) in addition to using a standardized data collection tool, such as validated questionnaires, to improve data comparability [24, 52].

The standardization of the definition of the terms “alternative, traditional or complementary medicine” or even the term used in PNS (Complementary and Integrative Practices) is also a factor that makes comparisons with international studies difficult. A limitation of the present study was asking only about the use of homeopathy, acupuncture and medicinal plants and herbal medicines. The option “others” could not be better detailed; however, the prevalence was low compared to the other CAM. A comprehensive systematic review observed that there is a variability through which CAM are defined and classified in the studies [24]. When comparing studies from different countries, the designation of complementary practices ranged from 4 to 36 therapies [24]. Another limitation was related to the use of the terms “medicinal plants” and “herbal medicines”: according to WHO document, published in 2013, they have different meanings [53] but they were included in the same question in the research questionnaire.

The strength of the study is the fact that it is the first publication that included data about the prevalence of CAM use in a representative survey of the Brazilian population.

## Conclusions

Despite the efforts made by the Brazilian Ministry of Health in recent years to implement the National Policy for CAM in Brazil and expand the number of therapeutic practices included in it, the Brazilian adult population has not been using these therapies. Therefore, it is recommended that the Ministry of Health invest in professional training in CAM, structuring these practices into services, expanding access to CAM for SUS users, and improving the recording of information about these practices. Moreover, the encouragement of evidence-based research and studies on the safety of herbal medicines and complementary practices in medicine are required, as well as questions related to the conservation of medicinal plants and intellectual property rights.

## Data Availability

The dataset generated and analysed during the current study are publicly available at the Instituto Brasileiro de Geografia e Estatística (IBGE) permanent website: https://www.ibge.gov.br/estatisticas/sociais/justica-e-seguranca/9160-pesquisa-nacional-de-saude.html?=&t=downloads

## Abbreviations

CAM: Complementary and Alternative Medicine
IBGE: Instituto Brasileiro de Geografia e Estatística (Brazilian Institute of Geography and Statistics)
PIC: Práticas Integrativas e Complementares em Saúde (Complementary and Integrative Practices)
PNPIC: Política Nacional de Práticas Integrativas e Complementares (National Policy for Complementary and Integrative Practices)
PNS: Pesquisa Nacional de Saúde (National Health Survey)
SUS: Sistema Único de Saúde (Brazilian Unified Health System)
WHO: World Health Organization

## References

[1] Bulletin of the World Health Organization (1998). Regulatory situation of herbal medicines.

[2] Onyiapat JL, Okoronkwo IL, Ogbonnaya NP (2011). Complementary and alternative medicine use among adults in Enugu, Nigeria. BMC Complement Altern Med.

[3] Laelago T, Yohannes T, Lemango F (2016). Prevalence of herbal medicine use and associated factors among pregnant women attending antenatal care at public health facilities in Hossana town, Southern Ethiopia: facility based cross sectional study. Arch Public Health.

[4] Pearson H, Fleming T, Chhoun P, Tuot S, Brody C, Yi S (2018). Prevalence of and factors associated with utilization of herbal medicines among outpatients in primary health centers in Cambodia. BMC Complement Altern Med.

[5] Brazil. Ministry of Health. Health Care Secretariat. Department of Primary Health Care (2015). National policy for complementary and integrative practices in SUS: access extension attitude/ Ministry of Health. Health Care Secretariat. Department of Primary Health Care.

[6] WHO Meeting on the Promotion and Development of Traditional Medicine (1977: Geneva) & World Health Organization (1978). The promotion and development of traditional medicine : report of a WHO meeting report of a WHO meeting [held in Geneva from 28 November to 2 December 1977].

[7] World Health Organization (WHO) (2002). WHO Traditional Medicine Strategy 2002–2005.

[8] Peprah P, Agyemang-Duah W, Arthur-Holmes F, Budu HI, Abalo EM, Okwei R, Nyonyo J (2019). ‘We are nothing without herbs’: a story of herbal remedies use during pregnancy in rural Ghana. BMC Complement Altern Med.

[9] Barreto GE, Avila-Rodriguez M, Foitzick M, Aliev G, Echeverria V (2017). Advances in medicinal plants with effects on anxiety behavior associated to mental and HealthConditions. Curr Med Chem.

[10] MacPherson H, Vertosick EA, Foster NE, Lewith G, Linde K, Sherman KJ, Witt CM, Vickers AJ (2017). The persistence of the effects of acupuncture after a course of treatment: a meta-analysis of patients with chronic pain. Pain..

[11] Goyatá SLT, Avelino CCV, Santos SVM, Souza DI, Gurgel MDSL, Terra FS (2016). Effects from acupuncture in treating anxiety: integrative review. Rev Bras Enferm.

[12] MacLennan AH, Wilson DH, Taylor AW (1996). Prevalence and cost of alternative medicine in Australia. Lancet.

[13] Eisenberg DM, Davis RB, Ettner SL (1998). Trends inalternative medicine use in the United States, 1990–1997: results of a follow-up national survey. JAMA.

[14] Thomas K, Coleman P (2004). Use of complementary or alternative medicine in a general population in Great Britain. Results from the National Omnibus survey. J Public Health.

[15] Stickley A, Koyanagi A, Richardson E, Roberts B, Balabanova D, McKee M (2013). Prevalence and factors associated with the use of alternative (folk) medicine practitioners in 8 countries of the former Soviet Union. BMC Complement Altern Med.

[16] El-Nimr NA, Wahdan IM, Wahdan AM, Kotb RE (2015). Self-medication with drugs and complementary and alternative medicines in Alexandria, Egypt: prevalence, patterns and determinants. East Mediterr Health J.

[17] BRAZIL. Ministry of Health. National Policy for Complementary and Integrative Practices (2006). Ordinance n. 971.

[18] Brazil. Ministry of Health. Health Care Secretariat. Ordinance n. 702, of March 21 (2018). It alters the consolidation ordinance n. 2/GM/MS, of September 28, 2017, to include new practices into the National Policy for Complementary and Integrative Practices – PNPIC Brazilian Federal Register 2018.

[19] Brazil. Ministry of Health. Revista Brasileira Saúde da Família (2008). Complementary and Integrative Practices in Health: a reality in SUS.

[20] Souza-Junior PRB, Freitas MPS, Antonaci GA, Szwarcwald CL (2015). Desenho da amostra da Pesquisa Nacional de Saúde 2013. Epidemiol Serv Saude.

[21] Sistema Integrado de Pesquisas Domiciliares (SIPD). Ministério do Planejamento, Orçamento e Gestão / Instituto Brasileiro de Geografia e Estatística – IBGE (2013). Pesquisa Nacional de Saúde.

[22] Damacena GN, Szwarcwald CL, Malta DC, Souza-Junior PRB, Vieira MLFP, Pereira CA, Morais Neto OL, Silva Júnior JB (2015). O processo de desenvolvimento da Pesquisa Nacional de Saúde no Brasil, 2013. Epidemiol Serv Saude.

[23] ABEP - Associação Brasileira de Estudos Populacionais*.* Brazil Criterion Available at: http://www.abep.org/criterio-brasil. Accessed 21 Mar 2019.

[24] Harris PE, Cooper KL, Relton C, Thomas KJ (2012). Prevalence of complementary and alternative medicine (CAM) use by the general population: a systematic review and update. Int J Clin Pract.

[25] Barnes PM, Bloom B, Nahin RL (2008). Complementary and alternative medicine use among adults and children: United States, 2007. Natl Health Stat Report.

[26] Pokladnikova J, Selke-Krulichova I (2016). Prevalence of complementary and alternative medicine use in the general population in the Czech Republic. Forsch Komplementmed.

[27] World Health Organization (2019). WHO global report on traditional and complementary medicine 2019.

[28] Ock SM, Choi JY, Cha YS, Lee J, Chun MS, Huh CH, Lee SY, Lee SJ (2009). The use of complementary and alternative medicine in a general population in South Korea: results from a national survey in 2006. J Korean Med Sci.

[29] Yamashita H, Tsukayama H, Sugishita C (2002). Popularity of complementary and alternative medicine in Japan: a telephone survey. Complement Ther Med..

[30] Lim MK, Sadarangani P, Chan HL, Heng JY (2005). Complementary and alternative medicine use in multiracial Singapore. Complement Ther Med..

[31] Siti ZM, Tahir A, Farah AI (2009). Use of traditional and complementary medicine in Malaysia: a baseline study. Complement Ther Med.

[32] Tangkiatkumjai M, Boardman H, Walker DM (2014). Herbal and dietary supplement use in Bangkok: a survey. J Complement Integr Med.

[33] Xue CC, Zhang AL, Lin V (2007). Complementary and alternative medicine use in Australia: a national population-based survey. J Altern Complement Med.

[34] Wu CH, Wang CC, Tsai MT, Huang WT, Kennedy J (2014). Trend and pattern of herb and supplement use in the United States: results from the 2002, 2007, and 2012 national health interview surveys. Evid Based Complement Alternat Med.

[35] Steinsbekk A, Rise MB, Johnsen R (2011). Changes among male and female visitors to practitioners of complementary and alternative medicine in a large adult Norwegian population from 1997 to 2008 (the HUNT studies). BMC Complement Altern Med.

[36] Kristoffersen AE, Stub T, Salamonsen A, Musial F, Hamberg K (2014). Gender differences in prevalence and associations for use of CAM in a large population study. BMC Complement Altern Med.

[37] Tindle HA, Davis RB, Phillips RS, Eisenberg DM (2005). Trends in use of complementary and alternative medicine by US adults: 1997-2002. Altern Ther Health Med.

[38] Neiberg RH, Aickin M, Grzywacz JG, Lang W, Quandt SA, Bell RA, Arcury TA (2011). Occurrence and co-occurrence of types of complementary and alternative medicine use by age, gender, ethnicity, and education among adults in the United States: the 2002 National Health Interview Survey (NHIS). J Altern Complement Med.

[39] Almeida G, Sarti FM, Ferreira FF, Diaz MD, Campino AC (2013). Analysis of the evolution and determinants of income-related inequalities in the Brazilian health system, 1998–2008. Rev Panam Salud Publica.

[40] Boccolini CS, de Souza Junior PR (2016). Inequities in healthcare utilization: results of the Brazilian National Health Survey, 2013. Int J Equity Health.

[41] World Health Organization (WHO) (2013). WHO traditional medicine strategy: 2014-2023.

[42] Xue CC, Zhang AL, Lin V, Myers R, Polus B, Story DF (2008). Acupuncture, chiropractic and osteopathy use in Australia: a national population survey. BMC Public Health.

[43] World Health Organization - WHO. Acupuncture: review and analysis of reports on controlled clinical trials. Geneva: WHO Library Cataloguing-in-Publication Data; 2002.

[44] Neto JF, Faria AA, Figueiredo MF (2009). Complementary and alternative medicine: use in Montes Claros, Minas Gerais. Rev Assoc Med Bras (1992).

[45] Bodeker GC, Ong C, Grundy C, Burford G, Shein K (2005). WHO global atlas of traditional, complementary and alternative medicine. Text volume.

[46] Relton C, Cooper K, Viksveen P, Fibert P, Thomas K (2017). Prevalence of homeopathy use by the general population worldwide: a systematic review. Homeopathy..

[47] Klein SD, Torchetti L, Frei-Erb M, Wolf U (2015). Usage of complementary medicine in Switzerland: results of the Swiss health survey 2012 and development since 2007. PLoS One.

[48] Santos RL, Guimaraes GP, Nobre MSC, Portela AS (2011). Análise sobre a fitoterapia como prática integrativa no Sistema Único de Saúde. Rev bras plantas med.

[49] Lima CAB, Lima ARA, Mendonça CV, Lopes CV, Heck RM (2016). O uso das plantas medicinais e o papel da fé no cuidado familiar. Ver Gaúcha Enferm.

[50] Aziz Z, Tey NP (2009). Herbal medicines: prevalence and predictors of use among Malaysian adults. Complement Ther Med.

[51] Brazil. Ministry of Health. Health Care Secretariat. Department of Primary Care (2018). Manual for Implementation of Complementary and Integrative Practices in SUS / Ministry of Health, Health Care Secretariat, Department of Primary Care.

[52] Quandt SA, Verhoef MJ, Arcury TA (2009). Development of an international questionnaire to measure use of complementary and alternative medicine (ICAM-Q). J Altern Complement Med.

[53] WHO. Guidelines on Good Agricultural and collection practices (GACP) for medicinal plantas, vol. 1. Geneve: World Health Organization Publications; 2003.

